# Induction of osteogenic differentiation of bone marrow stromal cells on 3D polyester-based scaffolds solely by subphysiological fluidic stimulation in a laminar flow bioreactor

**DOI:** 10.1177/20417314211019375

**Published:** 2021-06-24

**Authors:** Shuntaro Yamada, Mohammed Ahmed Yassin, Thomas Schwarz, Jan Hansmann, Kamal Mustafa

**Affiliations:** 1 Department of Clinical Dentistry, Faculty of Medicine – Tissue engineering group, University of Bergen, Bergen, Norway; 2 Fraunhofer Institute for Silicate Research ISC, Translational Center Regenerative Therapies, Wurzburg, Bayern, Germany; 3 Chair of Tissue Engineering and Regenerative Medicine, University Hospital Würzburg, Germany; 4 Department Electrical Engineering, University of Applied Sciences Würzburg-Schweinfurt, Germany

**Keywords:** Bone tissue engineering, bioreactor, lamina flow, dynamic cell culture, osteogenic differentiation

## Abstract

The fatal determination of bone marrow mesenchymal stem/stromal cells (BMSC) is closely associated with mechano-environmental factors in addition to biochemical clues. The aim of this study was to induce osteogenesis in the absence of chemical stimuli using a custom-designed laminar flow bioreactor. BMSC were seeded onto synthetic microporous scaffolds and subjected to the subphysiological level of fluid flow for up to 21 days. During the perfusion, cell proliferation was significantly inhibited. There were also morphological changes, with F-actin polymerisation and upregulation of ROCK1. Notably, in BMSC subjected to flow, mRNA expression of osteogenic markers was significantly upregulated and RUNX2 was localised in the nuclei. Further, under perfusion, there was greater deposition of collagen type 1 and calcium onto the scaffolds. The results confirm that an appropriate level of fluid stimuli preconditions BMSC towards the osteoblastic lineage on 3D scaffolds in the absence of chemical stimulation, which highlights the utility of flow bioreactors in bone tissue engineering.

## Introduction

Bone has a limited capacity for spontaneous healing of critical defects caused by injury, inflammation or therapeutic resection.^1^ Currently, extensive surgical intervention is required to restore the structure and function, often accompanied with considerable postoperative complications.^2^ Therefore, there is an urgent need for tissue engineering strategies, based on biomaterials in combination with multipotent cells and/or growth factors as an alternative approach to regenerate bone.^3^ Three-dimensional (3D) microporous scaffolds, which mimic the structure of bone matrix, are necessitated to control the spatiotemporal distribution of cells and bioactive clues. A porous structure promotes the osteogenic differentiation of multipotent cells. Specifically, highly-porous structures, with pore sizes ranging from 100 to 600 μm, appear to facilitate cell adhesion, growth and mineral formation as well as blood vessels formation in vivo within the construct.^4^ One of technical challenges to the clinical applications of cell therapy with scaffolding is its size. In a cell-based tissue engineering approach, 3D scaffolds and multipotent cells are the core of the engineered construct.^5^ As the volume of the 3D scaffold increases, the cells become vulnerable to a lack of nutrient and gas supply, which leads to the deterioration of cell viability and multipotency.^6^

Perfusing culture medium through scaffolds homogenises nutritional supply while removing waste products.^7^ Various types of bioreactors have been developed for this purpose, including spinner flasks bioreactors, rotating wall vessel bioreactors and laminar flow bioreactors.^8^ Moreover, fluid flow influences cell behaviours, and therefore, these bioreactor systems might be used to regulate the growth and differentiation of progenitors mechanically.^9^ The use of flow bioreactors has attracted particular attention in bone tissue engineering because bone remodelling is closely related to fluid micromovement due to the slight bone deformation which occurs as a result of physical activity.^10^ On the other hand, it has been reported that mammalian cells are susceptible to fluid shear, and an inappropriate level of shear stress leads to cell death.^11^ The shear-induced damage is further accelerated by turbulent flow, which is more likely to be generated in spinner flask and rotating wall vessel bioreactors.^11,12^ Laminar flow bioreactors facilitate precise control of fluid pattern through engineered constructs, which is considered to be a less damaging and more predictable procedure.^13^ Moreover, laminar flow bioreactors provide uniform gas and nutrient supply within the construct with a relatively low shear rate.^6^ Therefore, the use of laminar flow bioreactors seems advantageous for controlling the fate of progenitors by fluidic stimuli.

In bone tissue engineering, several studies in 2D flow systems reported the mechanical induction of osteogenesis without the presence of osteogenic supplements, namely the combination of dexamethasone, β-glycerophosphate and ascorbic acid or bone morphogenetic protein 2 (BMP-2). For examples, as early as 1 h after perfusion, osteoblast precursors, MC3T3-E1, responded to shear stress at 2 Pa by upregulating a key transcription factor, Runt-related transcription factor 2 (RUNX2), for osteogenesis.^14^ Further, the upregulation of other osteogenic markers such as collagen type 1 (COL1), osteocalcin (OCN) and alkaline phosphatase (ALP) was observed after 3 days of continuous perfusion.^15^ Similarly, mesenchymal stem/stromal cells (MSC) were reported to undergo osteogenesis solely as a response to mechanical stimuli. Studies using human bone marrow-derived MSC reported that shear stress at 0.4–2.2 Pa increased the expression of BMP-2, bone sialoprotein (BSP), osteopontin (OPN) and ALP together with enhanced calcium deposition within 7 days.^16–18^ Further comparable results were reported in MSC isolated from rodents at 1.09 mPa–1.03 Pa.^19^ Compared with 2D models, however, there is only limited evidence on fluid flow-induced osteogenesis in the 3D environment. Human fetal osteoblasts, hFOB 1.19, subjected to cyclic fluid shear stress at 3.93 mPa for 28 days on functionalised polycaprolactone/hydroxyapatite scaffolds, exhibited increased ALP activity, extracellular matrix (ECM) formation and mineralisation.^20^ A few studies of MSC in a 3D environment have reported promotion as well as suppression of osteogenesis by fluid flow. However, most of these studies were conducted in the presence of either chemical supplements or osteoinductive biomaterials such as decellularised matrix constructs, ECM-coated or hydroxyapatite-laden scaffolds.^13,21–25^ The lack of concrete evidence supporting fluid flow-induced osteogenesis in 3D scaffolds may be also attributed to the complexity of 3D dynamic culture systems. A relatively complex bioreactor set-up is required to establish stable culture conditions such as systems for environmental monitoring and control. Moreover, the assessment of flow pattern in culture chambers within 3D constructs is challenging. Unlike the 2D environment, fluid effects are exerted as multidirectional shear force (i.e. a sliding force applied parallelly to the surface) and pressure (i.e. a compressive force applied perpendicularly onto the surface). Therefore, it is computationally expensive to estimate fluid effects, and a thorough parameterisation of experimental configurations is necessary to achieve high predictive power.^26^

Thus, it remains unclear whether MSC osteogenesis can be induced solely by fluid flow in 3D constructs, namely, without the presence of the osteogenic supplements. The aim of the present study was to precondition MSC for bone regeneration by developing and optimising a laminar flow bioreactor. It was hypothesised that appropriate fluid flow could direct MSC towards the osteoblastic lineage in the absence of osteoinductive supplements/materials. A further aim was to determine the optimal flow for supporting cell growth while robust osteogenesis was induced. Bone marrow-derived MSC from Lewis rats (rBMSC) were seeded onto the 3D microporous scaffolds of synthetic copolymer, poly(L-lactide-co-trimethylene carbonate) lactide (LTMC), and subjected to fluid flow at different flow rates for 21 days in a custom-designed laminar flow bioreactor. Here, we successfully provoked osteogenesis in the bioreactor without the presence of osteogenic supplements, which was confirmed by their expression patterns of osteogenic and multipotent markers, cell proliferation, morphology, ECM formation and calcium deposition. The approach will further open the possibility of clinical translation of a laminar flow bioreactor as the induction of osteogenesis without using the chemicals could reduce concern over the risk of unforeseen effects of the drugs, and it is expected to enhance bone regeneration after the transplantation of preconditioned constructs into damaged sites.

## Materials and methods

### rBMSC isolation and expansion

The study was approved by the Norwegian Animal Research Authority (local approval number 20146866) and conducted according to the European Convention for the Protection of Vertebrates used for Scientific Purposes.

BMSC were isolated from the femurs of Lewis rats as described previously.^27^ The cells were maintained in alpha minimum essential medium (α-MEM: 22571-020, Gibco™, USA) supplemented with 10% fatal bovine serum (FBS: 10270-106, Gibco™, USA) and 1% penicillin and streptomycin (SV30010, HyClone, USA) at 37°C in 5% CO_2_ humidified atmosphere. rBMSC from the third and fourth passages were used in the experiments.

### Fabrication of 3D microporous scaffolds of LTMC and cell seeding

3D microporous scaffolds of LTMC, 1.2 mm in thickness and 12 mm in diameter, were fabricated by a salt particles-leaching technique as described previously.^28^ Briefly, LTMC (RESOMER^®^ LT706 S, Evonik) was dissolved in chloroform on a magnetic stirrer and mixed in petri dished with sodium chloride particles (diameter range 90–600 μm). A lid was left on to allow gradual evaporation of the chloroform. The dried construct was then punched into 12 mm diameter pieces and washed thoroughly with distilled water to remove the sodium chloride. The scaffolds were placed in 48 well plates and sterilised using 70% ethanol and ultraviolet exposure for 2 h. The characterisation of LTCM scaffolds was described previously.^29^

Before cell seeding, the scaffolds were prewetted with α-MEM for 24 h, and 250,000 cells were then seeded per scaffold and incubated at 37°C in 5% CO_2_ humidified atmosphere for 72 h, before being transferred into the bioreactor.

### Characterisation of rBMSC by multilineage differentiation and flow cytometry

To confirm the multipotency of rBMSC, their capacity for differentiation into osteoblasts, adipocytes and chondrocytes was tested. For osteogenic differentiation, rBMSC were seeded and incubated in α-MEM supplemented with 1% penicillin and streptomycin, 10% FBS, 173 μM L-ascorbic acid (A8960; Sigma-Aldrich, USA), 10 nM Dexamethasone (D4902; Sigma-Aldrich, USA) and 10 mM β-Glycerophosphate (G9422; Sigma-Aldrich, USA) for 21 days. To evaluate mineral deposition, the cells were stained with 0.1% Alizarin red S for 20 min at RT, followed by washing five times with Milli-Q water. For adipogenic differentiation, rBMSC were incubated in α-MEM supplemented with 1% Penicillin-Streptomycin, 10% FBS, 100 nM Dexamethasone, 10 μg/ml Insulin (I9278-5ML; Sigma-Aldrich, USA), 0.2 mM Indomethacin (17378 -5G; Sigma-Aldrich, USA) and 0.5 mM 3-Isobutyl-1-methylxanthine (I5879-250MG; Sigma-Aldrich, USA) for 14 days. To detect lipids, the cells were stained with 0.5% Oil Red O (CAT NO) in isopropanol for 30 min at RT, followed by washing three times with PBS. The cell nuclei were counterstained with Haematoxylin solution (GHS3-50 ml, Sigma-Aldrich, USA) for 5 min and washed three times with PBS. For chondrogenic differentiation, the cells were cultured in 3D pellet and micromass culture systems. Both culture methods used chondrogenic differentiation medium (CCM000/CCM020, R&D Systems, USA) in accordance with the manufacturer’s protocol. After 21 days of incubation, the chondrogenic pellets were embedded in Tissue-Tek^®^ O.C.T.™ Compound (4583, Sakura, Netherlands) and sectioned into 7 μm thick slices at −18°C using a cryostat (MNT, SLEE, Germany). The samples were stained with 1% Alcian Blue (pH 2.5; A5268, Sigma-Aldrich, USA) dissolved in acetic acid for 30 min at RT and washed five times with Milli-Q water.

For flow cytometry, rBMSC at passage three were trypsinised and resuspended in blocking buffer which consists of staining buffer (BUF0730, Bio Rad) with 0.5% BSA (37,525, ThermoScientific) and 2% FBS. After 1 h of incubation at 4°C, approximately 15,000–20,000 cells/staining were incubated with either primary antibodies or isotype controls for 30 min at 4°C in the dark. Primary antibodies and isotype controls used were anti-CD44H IgG2aκ antibody (1:100; 203901, BioLegend, USA), anti-CD73 IgG1κ antibody (1:100; 551123, BD Pharmingen, USA), PE anti-CD90 IgG1κ antibody (1:100; 551401, BD bioscience, USA), anti-Sca1/Ly6 polyclonal antibody (1:500; AB4336, Sigma-Aldrich, USA), FITC anti-CD34 IgG2aκ antibody (1:100; 11-0341-82, eBioscience, USA), PE anti-CD45 IgG1κantibody (1:100; 202207, BioLegend, USA), PE anti-CD79 IgG1κ antibody (1:100; 12-0792-41, eBioscience, USA), anti-Stro1 antibody (1:100; 14-6688-82, Invitrogen, USA), FITC Mouse IgG2aκ Isotype Ctrl Antibody (1:100; 400207, BioLegend, USA), PE Mouse IgG1κ Isotype Ctrl Antibody (1:100; 400111, BioLegend, USA), Mouse IgM Isotype Ctrl Antibody (1:100; 14-4752, Invitrogen, USA), Mouse IgG1κ Isotype Ctrl Antibody (1:100; 554121, BD Pharmingen, USA) and Mouse IgG2a Isotype Ctrl Antibody (1:100; 401501, BioLegend, USA). After the primary antibody incubation, the cells were sequentially centrifuged at 300 rcf for 5 min and washed three times with the staining buffer with 0.5% BSA. For the samples stained with unconjugated primary antibodies, the cells were subsequently incubated with secondary antibodies followed by sequential centrifugation and wash three times. Secondary antibodies used were Alexa Fluor 488 anti-mouse IgG antibody (1:500, A-11001, Invitrogen, USA), Alexa Fluor 488 anti-rabbit IgG antibody (1:500, A-11008, Invitrogen, USA) and Alexa Fluor 647 anti-mouse IgM antibody (1:500; A-21238, Invitrogen, USA). The data of the stained cells were captured by AccuriC6 (BD Biosciences, USA) and analysed using FlowJo software version 10.6.2 (Becton, Dickinson & Company, USA)

### Configuration of the lamina flow bioreactor and dynamic cell culture

**Figure 1.**
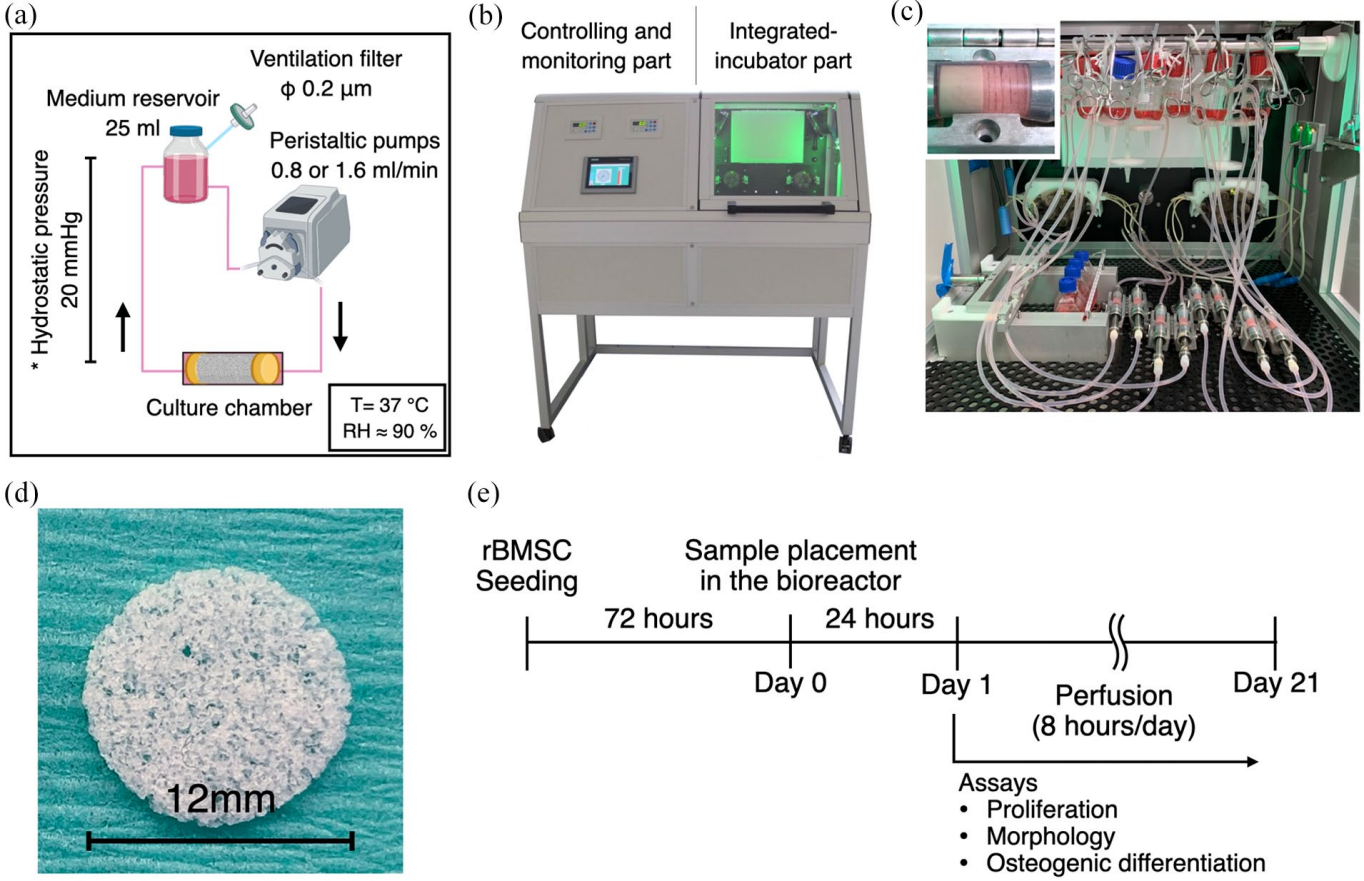
Experimental setup and timeline using the laminar flow bioreactor: (a) schematic illustration of environmental factors during dynamic culture. T: temperature, RH: Relative humidity, (b) the custom-made laminar flow bioreactor, which consists of controlling/monitoring part and integrated-incubation part, (c) image of inside-bioreactor during experiment. Culture chambers were connected to medium reservoirs via silicon tube, and medium was perfused by peristaltic pumps. In the culture chambers, 6 scaffolds (1.2 mm thickness and 12 mm diameter) were piled and sandwiched by rectifiers. The samples for the static control were also placed in the bioreactor so that they were subjected to the same environmental conditions except for fluid flow, (d) the optical image of the scaffold used. The scaffold was made of poly(L-lactide-cotrimethylene carbonate) and possess a microporous structure, and (e) experimental timeline. rBMSC were seeded on the scaffolds, and 3 days later, they were transferred into the bioreactor. Medium perfusion was performed for 8 h a day for 21 days.

The schematic experimental configuration is shown in Figure 1(a). The custom-designed laminar flow bioreactor was developed in the Fraunhofer Institute for Silicate Research. The bioreactor is equipped with 2 peristaltic pumps, monitoring sensors (temperature, gas and pressure), a CO_2_ injector, electric fans, a heating pad and a control panel (Figure 1(b)). To establish continuous perfusion, medium reservoirs and culture chambers were connected by silicon tubes (inner diameter 3.2 mm). The medium reservoirs were located approximately 30 cm above the culture chamber to suppress air bubble formation in the micropores. In the culture chambers, six scaffolds were stacked to give a total thickness of 7.2 mm and sandwiched by flow rectifiers (Figure 1(c) and (d)). Culture medium was perfused at either 0.8 (FL-L) or 1.6 ml/min (FL-H) for 8 h a day for 21 days (Figure 1(e)). The static control samples were placed in T25 flasks and incubated in the bioreactor so that both groups were subjected to the same environmental conditions (i.e. temperature, humidity, gas concentration and environmental frustration caused by opening/closing the bioreactor door). The same amount of culture medium, 25 ml, was supplied to each group for the consideration of paracrine effects, and half of the culture medium was refreshed every 3–4 days. The experiment was repeated six times to complete the planned assays.

### In-silico modelling of fluid flow and characterisation

For the estimation of fluid flow pattern and its mechanical effects, in silico modelling was undertaken using COMSOL Multiphysics version 5.6 (COMSOL AB, Sweden). Briefly, the geometry of the culture chamber including the scaffolds and the rectifiers was reproduced computationally. The mechanical properties of the culture medium were assumed to be comparable with water. The scaffolds were assigned as porous domains so that the Darcy’s law was applied to avoid excessive computational burden. Porous properties were parameterised using data previously obtained by microCT analysis of the scaffold, including porosity 91.708% and permeability 5.80216e-09 m^2^.^29^ From the inlet to the outlet, a fully developed flow was applied at a flow rate of either 0.8 ml/min (FL-L) or 1.6 ml/min (FL-H). Non-slip wall condition was applied to the boundary condition of the fluid paths (i.e. metal parts, silicon tubes). As a representative for visualisation of shear stress within the complex 3D structure, the geometry of the scaffold was obtained by microCT (Skyscan 1172, SkyScan, Belgium) primarily and converted into an .*stl* file. A cube with a diameter of 1.2 mm was dissected from the middle of the geometry to make it possible to proceed with computation. The dissected part was assumed to be located at the front row of the scaffold pile.

### Quantification of double strand DNA (dsDNA)

For the cell proliferation assay, the six samples were collected on day 3, 7, 14 and 21, in 0.1% Triton-X in Milli-Q^®^ water, and cell lysate was obtained by three freeze-thaw cycles. The experiment was repeated twice. The amount of dsDNA was quantified using Quant-iT™ PicoGreen™ dsDNA Assay Kit (P7589; Invitrogen, USA) in accordance with the manufacturer’s protocol. The intensity of fluorescence was measured at Ex/Em = 480/520 nm using a microplate reader (VLBL00D0; ThermoFisher Scientific, Finland).

### Quantification of ALP activity

The cell lysate was obtained by the same method described in the section 2-6. The 50 μl of cell lysate was incubated with the same amount of P-nitrophenyl phosphate (pNPP, 20-106; Sigma-Aldrich, Germany) for 5 min at room temperature. Absorbance was measured at 405 nm using the microplate reader.

### Quantitative reverse transcription polymerase chain reaction (RT-qPCR)

The samples for RT-qPCR were snap-frozen in liquid nitrogen on day 3, 7, 14 and 21 day and stored at −80°C until processed. Six scaffolds from one culture chamber were grouped into 3: 1st–2nd, 3rd–4th and 5th–6th scaffolds from the inlet, to examine variation within the group. Total RNA was extracted using a Maxwell^®^ 16 Cell LEV Total RNA Purification Kit (AS1280; Promega, USA) in accordance with the manufacturer’s protocol. Subsequently, reverse transcription was performed using a Transcription Kit (4368814; Applied Biosystems, USA). RT-qPCR was conducted with the StepOne™ real-time PCR system (4453320, Applied Biosystems, USA). The primers used are listed in table S1. Relative expression of each mRNA was calculated with the ΔΔCt method normalised by GAPDH.^30^ The data is represented as a mean value (±s.e.m) of three replicates.

### Immunofluorescent staining and confocal microscopy

The samples for immunofluorescence were obtained from the middle part of the piled scaffolds (i.e. third and fourth from the inlet) and fixed in 4% PFA for 15 min at room temperature unless otherwise stated. The samples were then permeabilised in 0.1% Triton X-100 in PBS (PBSTx) for 15 min at room temperature. Nonspecific binding was blocked with 20% normal goat serum in 0.1% Tween-20 in PBS (PBSTw) followed by incubation with primary antibodies overnight at 4°C. The primary antibodies used were anti-αTubulin antibody (1:250; 62204, Invitrogen, USA), anti-ROCK1 antibody (1:250; GTX113266, GeneTex, USA), anti-PCNA antibody (1:100; sc-56, Santa Cruz Biotechnology, USA), anti-RUNX2 antibody (1:250; ab192256, Abcam, UK) and anti-collagen type 1 antibody (1:1000; ab90395, Abcam, UK). Subsequently, the samples were washed five times, for 5 min each, with PBSTw. Incubation with secondary antibodies was undertaken with Phalloidin Alexa Fluor 488 (1:250, A12379; Invitrogen, USA) and 4′,6-diamidino-2-phenylindole (DAPI; 1:5000, 62247; Thermo Fisher Scientific, USA) for 1 h at RT followed by washing five times, for 5 min each, with PBSTw. The secondary antibodies used were Alexa Fluor 568 anti-rabbit IgG antibody (1:500; A11011, Invitrogen, USA) and Alexa Fluor 635 anti-mouse IgG antibody (1:500; A31575, Invitrogen, USA). For collagen type 1 staining, ice-cold methanol was used as a fixative. For PCNA staining, antigen retrieval was performed with 10 mM sodium citrate (pH 6.0) at 95°C for 20 min prior to primary antibody incubation. For image acquisition, the samples were placed on confocal dishes and mounted in ProLong™ Gold antifade reagent (P36939; Invitrogen, USA). Z-Stack images were acquired by a confocal microscope (TCS SP8; Leica, Germany) equipped with 20x and 40x water immersion objectives. Images were processed and analysed with Fiji/ImageJ.^31^ All images were represented as *z*-stack images of 150 μm thickness.

### Alizarin red S staining and quantification

The six samples were fixed in 4% paraformaldehyde for 40 min and washed three times in Milli-Q^®^ water. Calcium deposition was evaluated by Alizarin Red S staining (0.1% Alizarin Red S, A5533; Sigma-Aldrich, USA) for 20 min followed by washing six times with Milli-Q^®^ water. For quantification, the dye was extracted with 100 mM cetylpyridium chloride overnight at room temperature. Absorbance was measured at 540 nm using the microplate reader.

### Statistics

All data are represented as mean ± standard deviation unless otherwise specified. For multiple comparison, the data were evaluated by one-way ANOVA followed by Bonferroni’s multiple comparisons test by using SPSS^®^ Statistics version 25 (IBM, USA). A *p* value <0.05 was considered to be statistically significant.

## Results

### Characterisation of rBMSC used in the study

**Figure 2.**
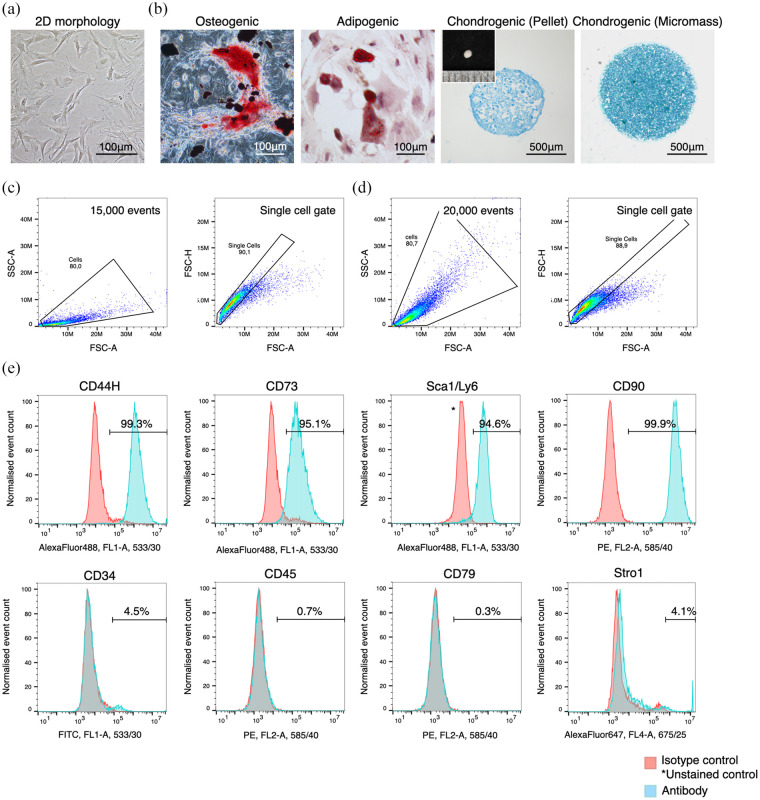
Characterisation of rBMSC used in the study: (a) rBMSC used in the study possessed a plastic adherent property and showed spindle morphology, (b) rBMSC were capable of differentiating into osteoblasts, adipocytes and chondrocytes under inductive culture conditions, (c) the gating strategy for the flow cytometry analysis for live cells and (d) fixed cells. Cells were distinguished from debris in the FSC-A VS SSC-A plots and then singlets were distinguished in the FSC-A VS FCS-H plot, and (e) rBMSC exclusively expressed putative rat MSC markers including CD44H, CD73, Sca-1/Ly6 and CD90 while they did not express haematopoietic markers including CD34, CD45 and CD79. Stro1 expression was only found in approximately 4% of the population.

rBMSC were characterised by their ability to adhere to plastic surfaces, multi-lineage differentiation and their expression of surface markers. The isolated cells were able to adhere to plastic surfaces, self-renew and differentiate into osteoblasts, adipocytes and chondrocytes under the inductive conditions (Figure 2(a) and (b)). Flow cytometry confirmed that the cells expressed putative rodent MSC markers including CD44H, CD73, Stem cell antigen-1 (Sca1/Ly6) and CD90, while little expression of haematopoietic markers, including CD34, CD45 and CD79, was observed. 4.1% of cells were identified as Stro-1 positive cells (Figure 2(c)–(e)).

### In silico modelling for flow characterisation

**Figure 3.**
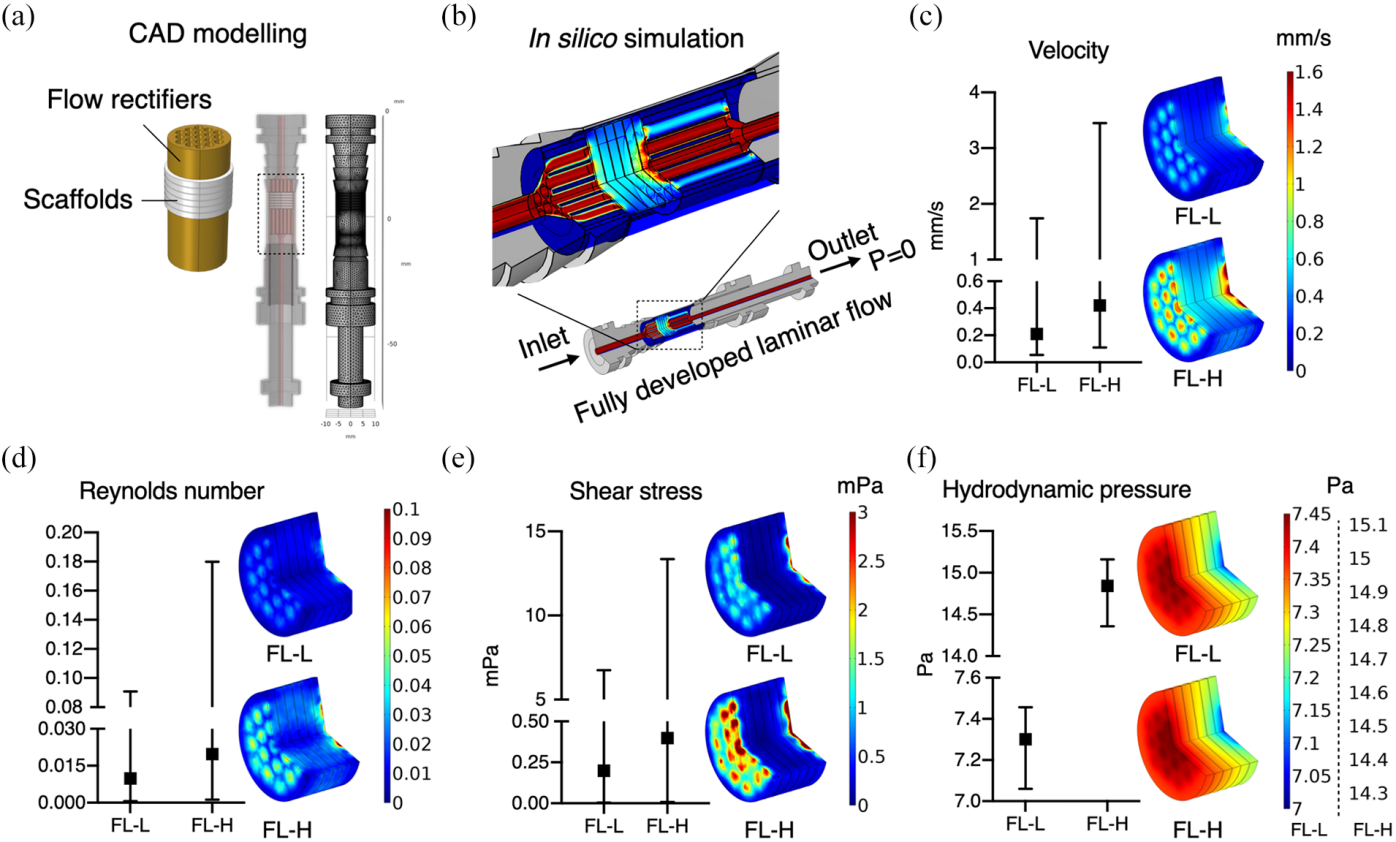
(a) The geometry of the culture chamber and the inlet/outlet was computationally reproduced. In the culture chambers, the scaffolds and rectifiers were placed as those in the actual experimental setting, (b) from the inlet, fully developed laminar flow of 0.8 ml/min (FL-L) or 1.6 ml/min (FL-H) was applied, and (c–f) the mean values and the range of velocity, Reynolds number, share stress, hydrodynamic pressure and their distribution within the scaffold construct were depicted.

**Figure 4.**
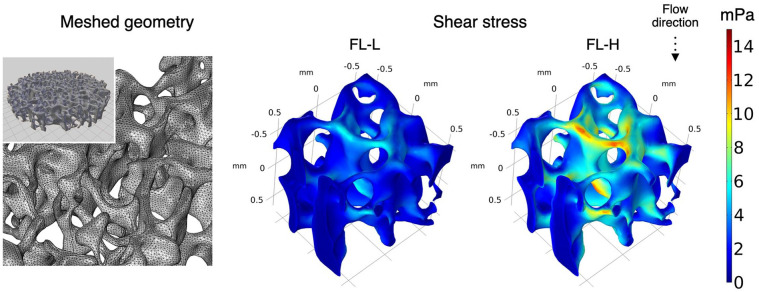
In silico modelling using the actual scaffold geometry obtained by microCT to illustrate the distribution of shear stress within the scaffold, the scaffold geometry was obtained by microCT and imported to the processing softwires as stl. file. To reduce computational burden, 1.2 mm cube was dissected from the middle of the scaffold and computed.

To estimate the characteristics and magnitude of fluid flow through the scaffold constructs in the bioreactor system, the culture chambers were reproduced computationally. In the chambers, stacked scaffolds with a total thickness of 7.2 mm were sandwiched by flow rectifiers and placed in the culture chamber (Figure 3(a)). From the inlet, fully developed laminar flow was applied (Figure 3(b)). The estimated mean values of fluid velocity within the scaffolds were 0.21 ml/min and 0.42 ml/min in FL-L and FL-H, respectively, despite a large local variation within the groups. The velocity was larger in the middle of the scaffold than in peripherical areas (Figure 3(c)). In both groups, the Reynolds number is below 0.2 at any point (Figure 3(d)). Shear stress distribution was corresponded with the local velocity, ranging from nearly 0 to 6.75 mPa (mean: 0.20 mPa) and to 13.35 mPa (mean: 0.40 mPa) in FL-L and FL-H, respectively (Figure 3(e)). The greatest hydrodynamic pressure (i.e. pressure exerted by fluid in motion) was estimated to occur on the scaffold at the inlet side, gradually decreasing as flow goes in, with a range from 7.06 to 7.46 Pa (mean: 7.30 Pa) and 14.36 to 15.16 Pa (mean: 14.84 Pa) in FL-L and FL-H, respectively (Figure 3(f)). The representative illustration of shear stress distribution within the actual geometry of the porous scaffold was given from the closest scaffold to the inlet (Figure 4). The magnitude of shear stress significantly varied from point to point for their complex 3D geometry, but overall, the magnitude of fluid effects from the simplified geometry with parameterisation seems comparable to the actual geometry.

### Inhibitory effect on proliferation and alteration of cell morphology/distribution under fluid flow

**Figure 5.**
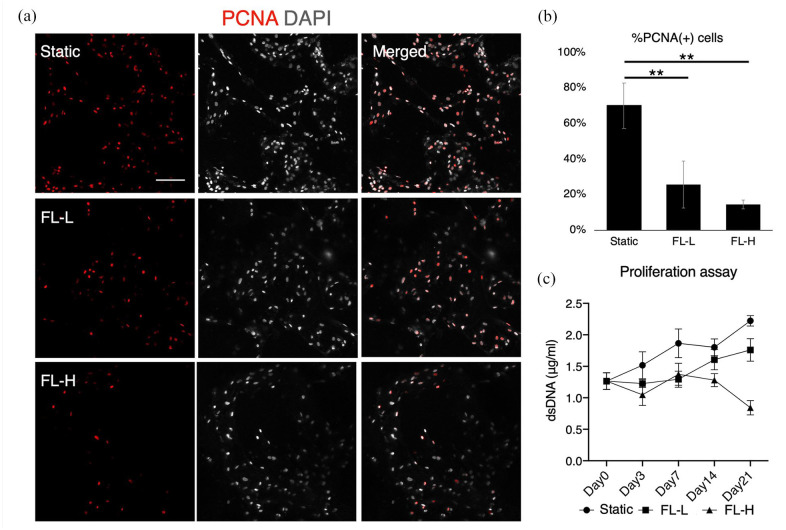
Proliferation of rBMSC under differential rate of fluid flow: (a) immunofluorescent images of a proliferation marker, PCNA, after 7 days of static culture and perfusion cultures at the flow rate of 0.8 ml/min (FL-L) and 1.6 ml/min (FL-H), (b) quantification of %PCNA positive cells, and (c) quantification of double-strand DNA (dsDNA) in the scaffold construct. While rBMSC highly proliferated during the period of 21 days in the static control, the cells in the FL-L and FL-H showed reduced proliferation capability. In the FL-H group, the amount of dsDNA decreased significantly on day 21. Scale bar = 100 μm. **p < 0.01.

**Figure 6.**
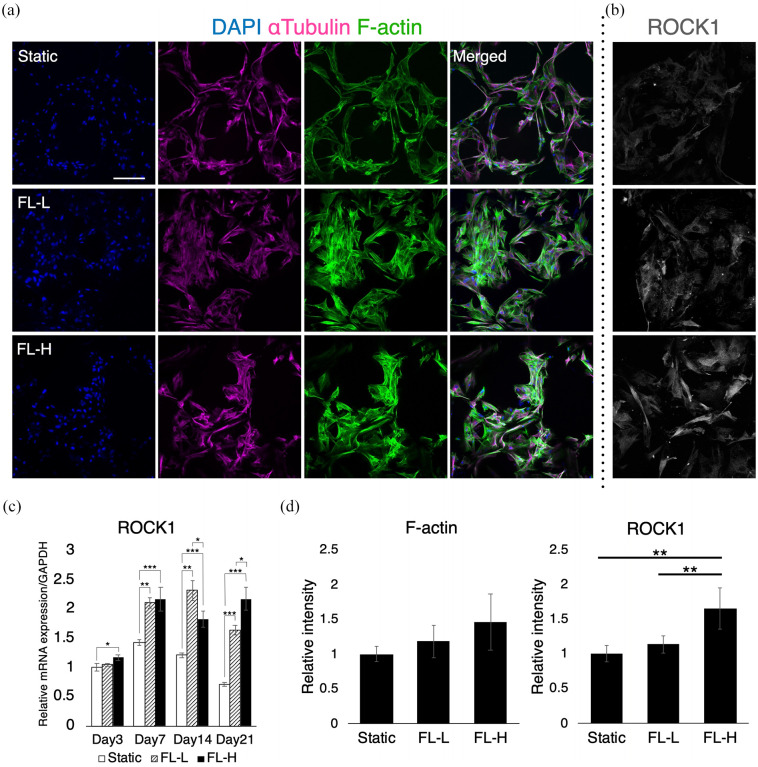
Morphological assessment of rBMSC subjected to differential flow rates for 7 days: (a and b) immunofluorescent images of αTubulin, F-actin and ROCK 1, (c) mRNA expression of ROCK1, and (d) image quantification of F-actin and ROCK1 intensity. Under the effect of fluid flow, rBMSC altered their morphology and alignment accompanied with the upregulation of ROCK1. FL-L: 0.8 ml/min, FL-H: 1.6 ml/min. Scale bar = 100 μm. *p < 0.05. **p < 0.01. ***p < 0.001.

To evaluate general cell behaviours under the fluid effects in a 3D environment, proliferation activity and cell morphology were assessed by immunofluorescence and the quantification of dsDNA. The expression of a proliferation marker, PCNA, shows that the majority of rBMSC in the static condition were highly proliferative on day 7, but PCNA positive cells decreased significantly under fluid flow, to approximately one-third compared with the static counterpart (FL-L: *p* = 0.007 and FL-H: *p* = 0.002) (Figure 5(a) and (b)). The percentage of PCNA positive cells was slightly higher in FL-L than in FL-H, but the difference was not statistically significant (*p* = 0.74). The inhibitory effect of fluid flow on proliferation was confirmed by the quantification of dsDNA, showing that the proliferation of rBMSC in FL-L fell below the static counterpart, and in FL-H, rBMSC did not increase but decrease over the period of 21 days (Figure 5(c)). No significant difference was observed in the amount of dsDNA among the stack of scaffolds within each group. In a static condition, rBMSC maintained their spindle shape and aligned uniformly along with the porous structure of the scaffolds on day 7 (Figure 6(a) and (b)). In contrast, rBMSC subjected to fluid flow tended to show more spreading morphology. Cell distribution was not as even, and the area of cell aggregation was locally observed both in FL-L and FL-H groups as the cells formed scattered colonies. The localisation of colony-like aggregates was spotted at both peripherical and middle regions of the scaffold throughout the stacked construct. Under perfusion, rBMSC showed higher intensity of F-actin and ROCK1. RT-qPCR and image quantification showed that the cells in FL-L and FL-H upregulated ROCK1 in mRNA and protein levels significantly (Figure 6(c) and (d)). ROCK1 intensity was significantly higher in the FL-L group and FL-H groups, and statistical significance was found in the static control versus FL-H (*p* = 0.0011) and FL-L versus FL-H (*p* = 0.0040). The mean intensity of F-actin also increased as the flow rate increased although there was no statistical significance.

### Fluid flow-induced osteogenesis of rBMSC in the absence of osteogenic supplements

**Figure 7.**
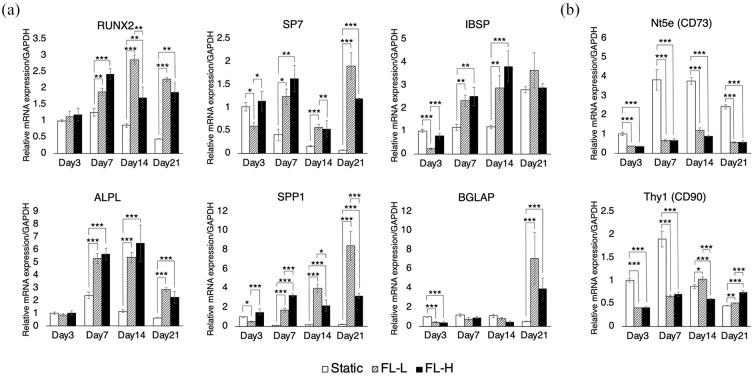
mRNA expression profile of osteogenic and putative MSC markers under perfusion by RT-qPCR: (a) rBMSC in the FL-L (0.8 ml/min) and FL-H (1.6 ml/min) groups significantly upregulated their early to middle osteogenic markers including RUNX2, SP7, IBSP, ALPL and SPP1 over the experimental period. A late osteogenic marker, BGLAP, was significantly upregulated in the dynamic culture groups on day 21. The upregulation was observed without the presence of osteogenic supplements and (b) putative MSC markers, CD73 and CD90, were significantly downregulated in the FL-L and FL-H. All data are displayed as mean ± s.e.m. *p < 0.05. **p < 0.01. ***p < 0.001.

During the dynamic culture, mRNA expression of osteogenic markers as well as putative MSC markers were evaluated by RT-qPCR (Figure 7(a) and (b)). A noticeable variation in the expression patterns of the evaluated mRNA was not found between 1st–2nd, 3rd–4th and 5th to 6th scaffolds. A key transcription factor for osteogenesis, RUNX2, was consistently upregulated in the dynamic culture groups. On day 7, the expression level was 1.5 times and 2.0 times higher in FL-L (*p* = 0.0023) and FL-H (*p* < 0.001), respectively. While expression in the static control decreased from day 7 to day 21, RUNX2 levels remained higher in the FL-L and FL-H groups. There was a similar tendency in the expression of another key transcription factor, SP7, which showed significant upregulation in both FL-L and FL-H groups from day 7 onwards compared with the static control. Furthermore, the upregulation of other osteogenic markers including IBSP, ALPL, and SPP1 was observed from day 7 onwards. A late marker of osteogenesis, BGLAP, was also upregulated by approximately 7.1 and 3.9 times in FL-L (*p* < 0.001) and FL-H (*p* < 0.001) groups, respectively, on day 21. The level of expression of these osteogenic markers was consistently higher in the FL-L than in the FL-H on day 21. It was of interest to note that the putative MSC markers Nt5e (CD73) was dramatically downregulated over the period of 21 days and Thy1 (CD90) for the first 7 days during the induction of osteogenesis by fluid flow.

**Figure 8.**
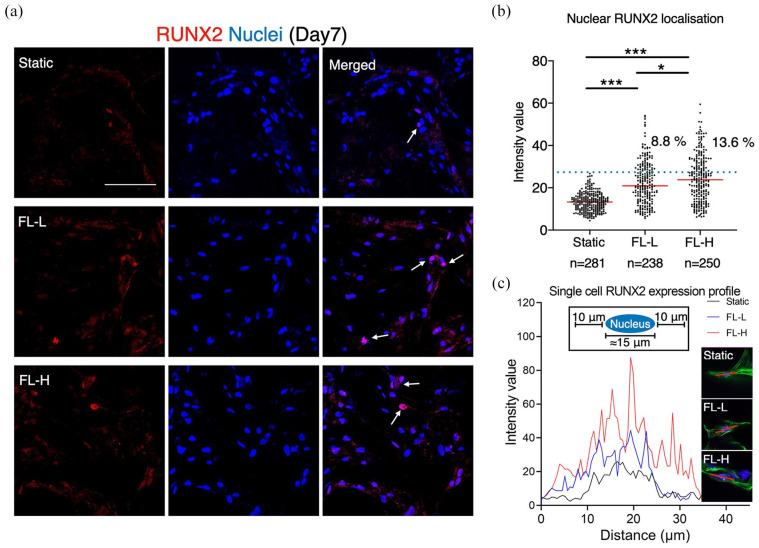
Upregulation and localisation of RUNX2 during the perfusion cell culture: (a) immunofluorescent images of RUNX2 showed that a small population of rBMSC in the FL-L (0.8 ml/min) and FL-H (1.6 ml/min) groups, but greater than the static counterpart, expressed RUNX2. Arrows indicate representative RUNX2 positive cells, (b) the image quantification revealed that 8.8% and 13.6% of the cells upregulated RUNX2 expression in the FL-L and FL-H, respectively, and (c) the line investigation using the representative cells confirmed that RUNX2 was localised in the nuclei but not in the intracellular areas. Scale bar = 100 μm. *p < 0.05. ***p < 0.001.

**Figure 9.**
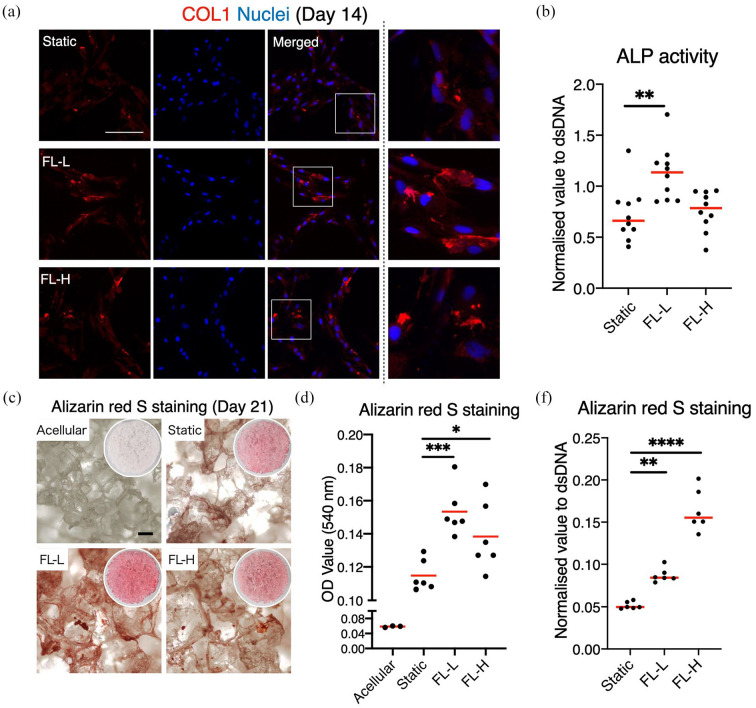
Promotion of rBMSC matrix production and calcium deposition by fluid flow: (a) immunofluorescent images of COL1 on day 14 showed that collagen formation was promoted in the FL-L (0.8 ml/min) and FL-H (1.6 ml/min) groups, (b) ALP activity was significantly higher on day 14 in the FL-L than the static counterpart and FL-H group, and (c–e) Alizarin red S staining on day 21 showed enhanced calcium deposition by rBMSC cultured under perfusion. Total amount of calcium deposition was the highest in the FL-L, but the ratio of the deposition per dsDNA was the highest in the FL-H. Scale bar = 100 μm. *p < 0.05. **p < 0.01. ***p < 0.001.

To confirm the upregulation of RUNX2 by fluid flow, the samples were analysed by immunofluorescence (Figure 8(a)). In the static condition, rBMSC scarcely expressed RUNX2. However, in the dynamic culture groups, some populations of rBMSC, but not the majority, presented localised RUNX2 in the nuclei on day 7. This was confirmed by the quantification of RUNX2, revealing that 8.8% and 13.6% of cell populations increased their levels of RUNX2 expression in the FL-L (*p* > 0.001) and FL-H (*p* > 0.001), respectively, (Figure 8(b)). The ratio of RUNX2 upregulated cells was higher in the FL-L than in FL-H (*p* = 0.032). Taking representative cells whose RUNX2 intensity level was defined as the mean (shown as red lines in Figure 8(b)), it was confirmed that RUNX2 was specifically localised in the nuclei but not in the intracellular region, and its intensity was highest in the FL-H group (Figure 8(b)). Osteogenic induction was further confirmed by the formation of collagen type 1, ALP activity, and mineral deposition. On day 14, fibrous collagen structure was detected in the dynamic culture groups, whereas only sporadic collagen was secreted in the static counterpart (Figure 9(a)). The collagen structure was more evident in FL-L than in the FL-H. ALP activity, which is necessary to calcify ECM, was significantly higher in the FL-L (*p* = 0.0080) (Figure 9(b)). On day 21, mineral deposition was assessed by Alizarin Red S staining. Although the level of deposition was low in all groups, the level of calcium in the scaffolds in the FL-L were higher than in the static control (*p* > 0.001) and FL-H (*p* = 0.036) (Figure 9(c) and (d)). However, once the value was normalised by dsDNA, mineral deposition per cell was greatest in the FL-H (Figure 6(e)).

## Discussion

Osteogenic preconditioning prior to transplantation was shown to enhance vascularization and bone formation at the recipient sites.^23,32^ Under clinical conditions, however, the chemical induction of osteogenesis (i.e. the application of dexamethasone or growth factors) may be contraindicated because of the risk of unforeseen effects. Dexamethasone is a synthetic glucocorticoid frequently supplemented both in adipogenic and in osteogenic media and determines the fate of MSC by regulating kye transcription factor for osteogenesis, RUNX2 and for adipogenesis, peroxisome proliferator-activated receptor γ2.^33^ The fetal determination depends on the maturity and density of progenitor cells, its concentration and synergetic effects with other regulatory molecules.^34^ Indeed, it has been suggested that the standard osteogenic medium may induce not only osteogenic but also adipogenic differentiation of BMSC simultaneously.^35,36^ Similarly, BMP-2 is an osteoinductive growth factor widely used to induce osteogenesis in vitro, and it is clinically approved by the U.S. Food and Drug Administration (FDA) for certain dental and orthoperiodic treatments to promote bone healing.^37^ However, a large number of severe adverse effects have been reported, which include postoperative inflammation, infection, ectopic ossification, osteolysis, arachnoiditis, neurological deficits, retrograde ejaculation and cancer.^38^ Therefore, various studies have attempted to induce osteogenesis in the absence of osteogenic supplements. These include the application of osteoinductive biomaterials such as ECM-coated synthetic polymers or hydroxyapatite and co-culture systems with endothelial cells or immune cells.^25,39^ Under natural conditions, bone homeostasis and remodelling are regulated by matrix strain and interstitial fluid movement caused by physical activity.^10,40^ Indeed, mechanical clues including the control of microstructure, surface stiffness and roughness of scaffolds, fluid shear stress, pressure, compression and stretching, are effective in stimulating osteogenic properties.^39,41–43^ In the present study, rBMSC were successfully preconditioned towards the osteoblastic lineage solely by applying low levels of fluid flow in the laminar flow bioreactor. Importantly, this was achieved without the presence of any type of osteoinductive chemicals/materials, by using medical-grade synthetic polymers, LTMC, as a scaffold material. The material offers biocompatibility and biodegradability suitable for bone regenerative therapy, but it is known to have low bioactivity and does not induce MSC osteogenesis alone.^44,45^ This excludes the possibility of a synergetic effect of bioactive (i.e. osteoinductive) components and fluid flow as previously described.^24,46,47^ Hence, the results provide evidence that osteogenesis of BMSC on 3D non-osteoinductive scaffolds can be induced solely by fluid flow in the absence of osteogenic supplements as reported in 2D systems where fluid effect is purely represented as shear stress.^14,15,19,20^

Various types of bioreactors have been developed for bone tissue engineering. Each of the systems has distinctive features, and therefore, the identification of experimental parameters is the first step in reconciling the experimental data. Multiple factors influence cell behaviours in addition to flow magnitude. It has been suggested that the volume of culture medium determines cell behaviour. A previous study using osteoblasts showed the reduction of mineralisation in a medium volume-dependent manner.^48^ Similarly, excessive use of medium in perfusion bioreactors may dilute paracrine/autocrine factors and prevent the activation of the downstream targets.^49^ Therefore, we used 25 ml culture medium, which is the minimum volume to maintain constant perfusion in our system. The initial seeding density was 250,000 cells per scaffold, and six scaffolds were placed in each culture chamber. This medium-to-cell ratio compares well with the standard static culture protocol provided by the manufacturer. In consideration of the effect of medium-to-cell ratio on cell growth, the same amount of culture medium was also applied to the static control, and the cells showed optimal growth. Another key factor taken into consideration was the prevention of air bubbles. These impede fluid flow, and bubbles entrapped within the scaffolds may adversely affect cell growth.^13,50^ Indeed, air bubbles are readily generated in perfusion bioreactors used for bone tissue engineering due to continuous flow, serum proteins acting as a surfactant and the microporous structure and hydrophobic nature of 3D scaffolds.^51–54^ Therefore, Henry’s law was applied to supress bubble formation completely. Namely, 20 mmHg (≈2.7 kPa) of hydrostatic pressure was applied simply by raising the medium reservoirs by approximately 30 cm. Previously, 100–300 kPa of hydrostatic pressure was proposed for promotion of osteogenic differentiation, but the hydrostatic pressure applied in the present study was considerably lower and therefore not considered critical. ^43,55^ As a key stimulus, the effect of fluid flow needs to be identified. Various studies have tested the utility of flow bioreactors, and mostly the flow rate was stated. However, flow rate itself does not represent the magnitude of fluid effects, and indeed, the magnitude varies significantly depending on the geometry of the culture chambers and the macro- and microstructures of the scaffolds. Therefore, to allow eligible comparison with other systems, the type of flow (i.e. laminar or turbulent flow), shear stress and hydrodynamic pressure should be standardised to represent the characteristics of fluid flow. However, because real-time monitoring of these factors requires extensive equipment and is mostly impractical,^56,57^ in silico finite element analysis is utilised to estimate flow characteristics computationally.^58^ In silico analysis showed that the culture medium was relatively-evenly distributed within the scaffold constructs in the present study. The Reynolds numbers within the constructs were exclusively below 1, indicating laminar, but not turbulent flow.

The magnitude of fluid shear and dynamic pressure as well as the duration of perfusion are important determinants of cell growth and fate.^18^ Therefore, the flow rate and duration were determined on initiation of this study. Continuous perfusion at 0.8 ml/min for 24 h did not support cell growth unlike that for 8 h, and a high magnitude flow (i.e. 3.2 ml/min) for 8 h a day caused pronounced damage (Figure S1). This is supported by a previous in silico study, demonstrating that the nutrient and gas supply become uniform within the scaffold constructs as flow rate increases, but that the high shear stress might cause cell death in vitro.^6^ Even if it does not cause cell death, fluid shear reduces cell proliferation of rBMSC in a dose-dependent manner by arresting the cell cycle at G0/G1.^59^ Therefore, in this study, relatively low flow rates were tested: 0.8 ml/min and 1.6 ml/min for 8 h per day up to 21 days. In the FL-L group, rBMSC were subjected to shear stress up to 6.75 mPa and hydrodynamic pressure up to 7.46 Pa. In the FL-H group, rBMSC were subjected to shear stress up to 13.35 mPa and hydrodynamic pressure up to 14.36 Pa. These values were presumably lower than shear stress and pressure exerted by interstitial flow in bone marrow under physiological as well as loaded conditions.^60–62^ As expected, even these levels of gentle fluid stimulus supressed cell growth compared with the static culture condition. Nevertheless, the data showed that FL-L allowed rBMSC to gradually increase in number while FL-H prevented cell growth. Morphological evaluation revealed that rBMSC under perfusion altered their alignment within the scaffold construct and formed colony-like aggregates. It is known that the formation of cell aggregates facilitates paracrine signalling and activates biological events including osteogenic differentiation.^63,64^ The alternation of cell morphology and alignment were accompanied with enhanced F-actin polymerisation and upregulation of ROCK1. ROCK1 plays a crucial role in cell motility, adhesion and cell contraction by activating actomyosin complex, and therefore, rBMSC presumably underwent self-reorganisation to adapt to the environment. Previous studies have shown that ROCK1 activity is associated with MSC proliferation and osteogenic differentiation.^65,66^ ROCK1 activity is negatively correlated with cell proliferation, while its activation promotes RUNX2 expression, leading to osteogenesis. The present study also suggests that ROCK1 activation caused by fluid flow may link to an inhibitory effect on proliferation and the promotion of osteogenesis. However, further studies are needed to confirm the causal relationship between fluid stimuli, ROCK1 activation and the induction of osteogenesis.

Previous studies using a 2D microfluidic system showed that sub-physiological shear stress was sufficient to upregulate osteogenic markers.^17,19^ Therefore, we further evaluated fluid-induced osteogenesis in a 3D environment where fluid effects were exerted not only as shear stress but also as pressure. In both FL-L and FL-H, the mRNA expression of early-to-mid phase osteogenic markers, RUNX2, SP7, IBSP, ALPL and SPP1 was consistently upregulated over the period of 21 days. Upregulation of a late osteogenic marker, BGLAP, was observed only in the FL-L and FL-H groups on day 21, clearly suggesting that fluid stimuli directed the fate of rBMSC towards the osteoblastic lineage. The immunofluorescence showed that approximately 10% of rBMSC in the dynamic culture groups upregulated their RUNX2 expression in protein level on day 7. It was localised to the nuclei, indicating that RUNX2 acted as a transcription factor. Noteworthily, although the mRNA expression of RUNX2 was upregulated as a whole construct (i.e. a stack of six scaffolds) in the dynamic culture groups from day 7 onwards, the immunofluorescence and the quantification confirmed that the expression pattern of RUNX2 in protein level was rather heterogeneous. This may be attributed to not only the heterogeneous flow pattern but also the heterogeneous population of rBMSC: some of the population seemed more prone to be directed towards the osteogenic lineage by the fluid stimuli. Furthermore, it has been reported that the mRNA and protein expression of RUNX2 does not always coordinate each other, and the localisation of RUNX2 in nuclei as a transcription factor is spatiotemporally regulated.^67^ In rBMSC, the consequence of the robust mRNA expression of RUNX2 may be either cytoskeletal expression/diffusion or nuclear localisation.^29^ In the present study, the quantification and line investigation confirmed that the mRNA upregulation of RUNX2 by fluid flow was accompanied by a degree of nuclear localisation. Similarly, collagen formation, ALP activity and mineral deposition were found to be enhanced in the dynamic culture groups. It is of interest to note that the FL-H group seemed to show higher osteogenic induction than the FL-L group despite the strong inhibitory effect on cell proliferation. This may accord with the tendency to gradually lose proliferative capability as they undergo differentiation.^68^ Nevertheless, the induction of osteogenesis, particularly in terms of collagen formation and mineral deposition, by fluid flow is apparently not as robust as that achieved by the osteogenic supplements as previously tested in a study of osteogenic differentiation of rBMSC on LTMC scaffolds.^29^ This could be attributable to the characteristics of each component of the common osteogenic supplement (i.e. dexamethasone, β-glycerophosphate and ascorbic acid). Dexamethasone is known to boost MSC proliferation before inducing differentiation, whereas in the present study the fluid flow caused simultaneous inhibition of proliferation.^69^ This resulted in a lower cell number than in the static control. β-glycerophosphate acts as a source of phosphate to produce calcium phosphate, and therefore relatively low mineral production may be attributable to a lack of phosphate. Ascorbic acid is essential for collagen synthesis. In the present study, α-MEM was used as a standard growth medium in the study. It contains 50 mg/l of ascorbic acid, and therefore collagen formation was observed in all the groups but enhanced by the fluid flow.

Apart from the mechanical stimuli from fluid flow, mass transport may have influenced cell behaviours. The previous in silico study demonstrated an increase in nutrient/gas diffusion within a 3D construct in a velocity-dependent manner.^6^ Indeed, the scaffolds placed in the static condition were subjected to reduced mass transport in comparison with the dynamic condition. This may have led to increased concentration gradient within the scaffolds: the highest concentration of O_2_ and nutrients at the surface and waste products at the core. Therefore, in theory, the mass transport in the static control largely depended on a passive diffusive flux driven by the concentration gradient, whereas it was predominantly by the medium movement in the dynamic condition. Nevertheless, in the present study, the highly porous scaffolds with porosity 91.708% and permeability 5.80216e-09 m^2^ were designed. This presumably allowed sufficient diffusion which supported optimal cell growth even without the presence of flow, and it was microscopically confirmed that cell distribution and proliferation profile were uniform among the scaffold even in the static condition. Admittedly, the effects of mechanical stimuli and mass transport would not be separable in the 3D culture system, but the effect of variance in mass transport could be considered minimum between the groups.

BMSC from Lewis rats were used in this study in order to avoid the risk of donor-to-donor variation. Hence, the data are not necessarily generalisable to BMSC isolated from other species including humans. Noteworthily, BMSC are more osteoblast-oriented as a nature, and fluid flow may therefore promote their spontaneous differentiation into the osteoblastic lineage.^70^ It was reported that adipose tissue-derived MSC (AT-MSC) were also mechanosensitive, and AT-MSC acquired osteoblastic nature after receiving fluidic mechanical stimulation in the presence of 1,25-dihydroxyvitamin D3, but whether fluid flow solely induces the osteogenesis of AT-MSC or other types of MSC apart from BMSC remains elusive.^71^ The optimal magnitude of fluid effects and the duration for the purpose of osteogenic induction may be species-specific and possibly be donor-specific. Similarly, the magnitude may vary considerably, depending on material properties such as the micro- and macro-geometry and surface chemistry of the scaffolds even when the same flow rate is applied. Further, bioreactor design must be taken into consideration. Flow rate therefore needs to be optimised for each experimental setting. Under the present study conditions, osteogenesis was successfully induced in a 3D environment in the laminar flow bioreactor in the absence of osteoinductive materials/supplements. The potential clinical implications have yet to be determined. Further in vivo study is warranted to translate the bioengineering technology into clinical application.

## Supplemental Material

sj-docx-1-tej-10.1177_20417314211019375 – Supplemental material for Induction of osteogenic differentiation of bone marrow stromal cells on 3D polyester-based scaffolds solely by subphysiological fluidic stimulation in a laminar flow bioreactor Click here for additional data file. Supplemental material, sj-docx-1-tej-10.1177_20417314211019375 for Induction of osteogenic differentiation of bone marrow stromal cells on 3D polyester-based scaffolds solely by subphysiological fluidic stimulation in a laminar flow bioreactor by Shuntaro Yamada, Mohammed Ahmed Yassin, Thomas Schwarz, Jan Hansmann and Kamal Mustafa in Journal of Tissue Engineering

sj-docx-2-tej-10.1177_20417314211019375 – Supplemental material for Induction of osteogenic differentiation of bone marrow stromal cells on 3D polyester-based scaffolds solely by subphysiological fluidic stimulation in a laminar flow bioreactor Click here for additional data file. Supplemental material, sj-docx-2-tej-10.1177_20417314211019375 for Induction of osteogenic differentiation of bone marrow stromal cells on 3D polyester-based scaffolds solely by subphysiological fluidic stimulation in a laminar flow bioreactor by Shuntaro Yamada, Mohammed Ahmed Yassin, Thomas Schwarz, Jan Hansmann and Kamal Mustafa in Journal of Tissue Engineering
