# Supplementary material for: Induction of osteogenic differentiation of bone marrow stromal cells on 3D polyester-based scaffolds solely by subphysiological fluidic stimulation in a laminar flow bioreactor
Source: J Tissue Eng. 2021 Jun 24;12:20417314211019375. doi: 10.1177/20417314211019375 (PMC8243246; doi:10.1177/20417314211019375)
Supplement: sj-docx-1-tej-10.1177_20417314211019375 – Supplemental material for Induction of osteogenic differentiation of bone marrow stromal cells on 3D polyester-based scaffolds solely by subphysiological fluidic stimulation in a laminar flow bioreactor [file sj-docx-1-tej-10.1177_20417314211019375.docx]

Table S1. Primers used for RT-qPCR gene expression analysis

| Gene | Symbol | Cat. No. |
| --- | --- | --- |
| Glyceraldehyde-3-phosphate dehydrogenase | GAPDH | Rn01749022_g1 |
| Rho-associated kinases 1  Runt-related transcription factor 2 | ROCK1  RUNX2 | Rn00681157_m1  Rn01512298_m1 |
| Sp7 transcription factor | SP7 (Osterix) | Rn01761789_m1 |
| Bone sialoprotein | IBSP | Rn00561414_m1 |
| Alkaline phosphatase | ALPL | Rn01516028_m1 |
| Secreted Phosphoprotein 1 (Osteopontin) | SPP1 | Rn00681031_m1 |
| Bone gamma-carboxyglutamate protein | BGLAP | Rn00566386_g1 |
| Ecto-5′-nucleotidase | Nt5e (CD73) | Rn00665212_m1 |
| Thy-1 | Thy1 (CD90) | Rn00562048_m1 |
