# Supplementary material for: Induction of osteogenic differentiation of bone marrow stromal cells on 3D polyester-based scaffolds solely by subphysiological fluidic stimulation in a laminar flow bioreactor
Source: J Tissue Eng. 2021 Jun 24;12:20417314211019375. doi: 10.1177/20417314211019375 (PMC8243246; doi:10.1177/20417314211019375)
Supplement: sj-docx-2-tej-10.1177_20417314211019375 – Supplemental material for Induction of osteogenic differentiation of bone marrow stromal cells on 3D polyester-based scaffolds solely by subphysiological fluidic stimulation in a laminar flow bioreactor [file sj-docx-2-tej-10.1177_20417314211019375.docx]

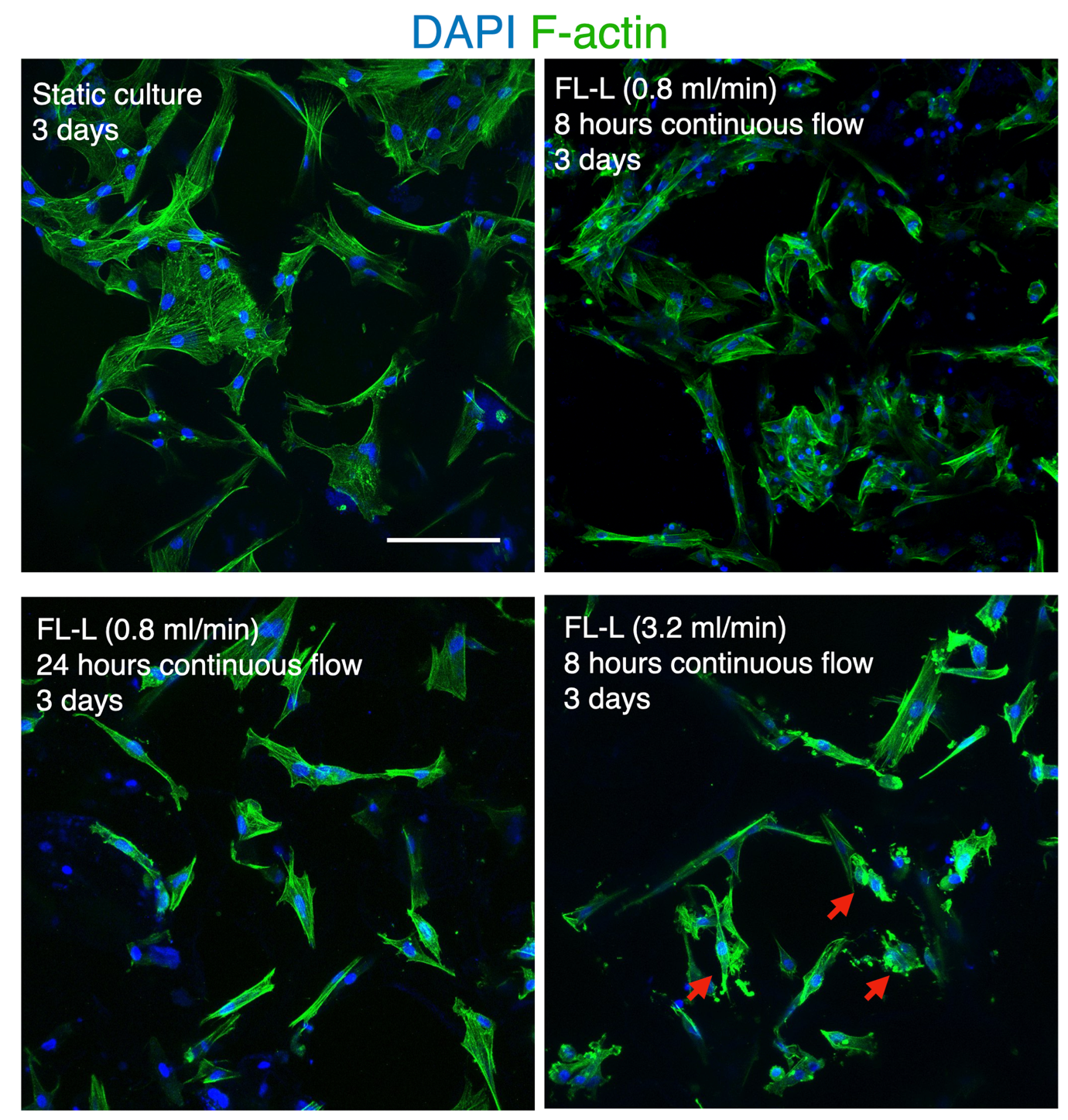


Fig. S1 Morphological change and cell damaging effect of differential flow rate and duration.

While rBMSC in the static condition as well as FL-L (i.e., 0.8 ml/min for 8 hours a day) exhibited an elongated morphology, 24 hours perfusion at 0.8 ml/min significantly suppressed cell elongation and cell-to-cell interaction. At flow rate of 3.2 ml/min, a greater number of apoptotic cells (indicated by arrows) and actin fragments due to cell collapse were observed.
